# Altered Functional Connectivity in an Aged Rat Model of Postoperative Cognitive Dysfunction: A Study Using Resting-State Functional MRI

**DOI:** 10.1371/journal.pone.0064820

**Published:** 2013-05-30

**Authors:** Peng Xie, Tian Yu, Xiaoyun Fu, Ye Tu, Yan Zou, Su Lui, Xuna Zhao, Xiaoqi Huang, Graham J. Kemp, Qiyong Gong

**Affiliations:** 1 Department of Anesthesiology, Zunyi Medical College, Zunyi, Guizhou, China, People's Republic; 2 The School of Public Health, Zunyi Medical College, Zunyi, Guizhou, China, People's Republic; 3 Huaxi MR Research Center (HMRRC), Department of Radiology, West China Hospital of Sichuan University, Chengdu, Sichuan, China, People's Republic; 4 Philips Medical Systems Beijing, Beijing, China, People's Republic; 5 Magnetic Resonance and Image Analysis Research Centre (MARIARC), Faculty of Health and Life Sciences, University of Liverpool, United Kingdom; Cuban Neuroscience Center, Cuba

## Abstract

**Background:**

Postoperative cognitive impairment is a common complication after cardiac and major non-cardiac surgery in the elderly, but its causes and mechanisms remain unclear. The purpose of the current study was to use resting-state functional magnetic resonance imaging (fMRI) to explore changes in the functional connectivity, i.e. the synchronization of low frequency fluctuation (LFF), in an animal model of cognitive impairment in aged rats.

**Methods:**

Aged (22 months) rats were anaesthetized with 40 µg/kg fentanyl and 500 µg/kg droperidol (intraperitoneal) for splenectomy. Cognitive function was assessed using Y maze prior to operation and on postoperative days 1, 3 and 9. To evaluate functional connectivity, resting-state fMRI data were acquired using a 3T MR imaging system with a 4 channel phase array rat head coil.

**Results:**

Cognitive function was impaired at postoperative days 1 and 3 compared with preoperative. Significant synchronized LFF was detected bilaterally in the primary somatosensory cortex and hippocampus preoperatively. By contrast, no significant LFF synchronization was detected in the right primary somatosensory cortex and right hippocampus on postoperative days 1 and 3, although the pattern of functional connectivity had become almost normal by day 9.

**Conclusion:**

Splenectomy performed under neuroleptic anaesthesia triggers a cognitive decline that is associated with altered spontaneous neuronal activity in the cortex and hippocampus.

## Introduction

Postoperative cognitive dysfunction (POCD) is the impairment of perception, memory, and information processing after surgery [1]. First described over 50 years ago [2], POCD is increasingly recognized [3]–[9] as a complication of non-cardiac [10] as well as cardiac surgery [11]. The mechanisms by which anaesthesia and surgery affect cognitive function are unknown, but risk factors for POCD include advanced age, long duration of surgery, and respiratory and infectious complications [12], [13]. A multicenter study found that POCD was present in 26% of patients 1 week and in 10% of patients 3 months after surgery [14]. POCD not only diminishes the quality of life, but also adds cost to hospitalization and out-of-hospital care [11], [15]. Thus the potential relationship between postoperative cognitive decline and anaesthesia and surgery merits more study.

Functional magnetic resonance imaging (fMRI) offers a useful perspective on brain function [16]–[20], and has recently been much used to study the brain at rest. Biswal and colleagues found that low frequency fluctuation (LFF) (<0.08 Hz) in the resting-state fMRI signal in the motor cortices showed synchronisation with a spatial pattern similar to the activation pattern of bilateral finger tapping [21]. Since then synchronised LFF has been demonstrated in motor, auditory, visual and sensorimotor cortical systems, among others [22]–[26]. Reflecting spatiotemporal correlations between spatially distinct regions of the brain, LFF are believed to measure functional connectivity [27]–[31]. Recent studies have shown decreased low-frequency correlations in the preclinical phase of mild cognitive impairment and Alzheimer's disease [32]–[34], raising the prospects that low-frequency functional connectivity may prove a useful noninvasive indicator of network function in neurodegenerative disease.

Postoperative cognitive decline has mainly been studied using either simple neuropsychological and behavioral methods [35], [36], or molecular and cellular approaches [6], [7], [37], [38]. Between these two levels, neuroimaging offers a way to visualize changes in cerebral function *in vivo* with high spatial resolution, and this is the approach we take here. Recently, spontaneous LFF in resting-state fMRI signals has been studied in the preclinical phase of neurodegenerative disease [39]–[41]. Here we applied this technique to a previously-described animal model of postoperative cognitive impairment [6] to ask whether there is a postoperative deficit in functional connectivity, and whether this is associated with cognitive dysfunction.

## Materials and Methods

### Animals and Surgical Operation

Female aged Sprague-Dawley rats (weight 400–550 g, mean age 22 months) were maintained in the temperature-, light/dark-controlled Animal Facilities of Chinese Academy of Science at Chengdu, China, with free access to rodent chow and water. Animal care was approved in accordance with the guidelines for Care and Use of Laboratory Animals in China.Splenectomy is chosen as a standardized organ removal intervention. Rats were anaesthetized with 40 µg/kg fentanyl and 500 µg/kg droperidol (intraperitoneal). A small incision was made in the left upper abdominal quadrant, the spleen was isolated, the arteries and veins ligated, the spleen removed and the wound closed by suture. Throughout surgery the animals were positioned over a heated pad: core temperature was monitored and maintained at 37–38°C, respiration rate at 80–100 breaths min^−1^ and oxygen saturation at 95–100% (Nellcor NBP-40, CO, USA). After recovery, animals were returned to their cages and housed individually. Surgical procedures were approved by the Institutional Animal Use and Care Committee of Chinese Academia of China at Chengdu.

### Cognitive Testing

Cognitive function was assessed as spatial memory using a Y maze prior to operation and on postoperative days 1, 3 and 9 according to the method developed and described in detail by Wan [6]. All training was performed in the morning, to habituate rats to the training environment. The Y maze consists of three arms (80×80×60 cm), with a light on the wall of each and the floor of the ‘stem’. One of the two branch arms has wires through which an electric shock can be applied; the other branch arm remains illuminated throughout.

### fMRI Study

fMRI was carried out prior to operation and on postoperative days 1, 3 and 9. For imaging, animals were anaesthetized with intraperitoneal chloral hydrate 300 mg/kg and placed in the scanner with an MR-compatible fixation device securing the head with a tooth bar. Body temperature was maintained at 37–38°C using warm air. fMRI data were acquired using a 3.0T MR imaging system (ACHIEVA, Philips, Netherlands) with a 4 channel phase array rat head coil. T_2_-weighted images were acquired using a 3D turbo spin echo sequence (TR/TE 2500/240 ms, slice thickness 1 mm, matrix size 224×224×30, flip angle 90°, FOV 50×50 mm^2^). Functional images were acquired using single shot spin echo EPI sequence (TR/TE 2000/27 ms, flip angle 90°, matrix size 96×96, FOV 50×41 mm^2^, thickness/gap 1/0 mm, a total of 120 volumes, 20 axial slices per volume to cover the whole brain). The paradigm consisted of 5 dummy scans to reach steady state followed by 120 scans during rest, for a total experiment time of 225 seconds.

To monitor physiological responses, the femoral artery was cannulated (n = 6), and samples of arterial blood taken at the beginning and end of the scanning for measurement of pH, *p*CO_2_ and *p*O_2_ using a blood gas analyzer (GEM Premier-3000, Instrumentation Laboratory, MA, USA).

### Data Analysis

Image data was preprocessed using statistical parameter mapping (SPM2, http: //www.fil.ion.ucl.ac.uk/spm/software/) including slice timing, head-motion correction, spatial normalization to a standard rat brain template [42] and smoothing with FWHM of 7.8 mm; all data was scaled by a factor of 10 for analysis. Further analyses were performed using MarsBar software (http: //*marsbar*.sourceforge.net/), including low-pass filtering, seed region identification and generation of correlation (functional connectivity) maps. Resting state time courses were first low-pass filtered with 0.08 Hz cutoff [21], [22]. Based on the standard rat brain template, spatially aligned to the atlas of Paxinos [43], seed regions of interest (ROIs) (2×2 pixels) were selected in the left primary somatosensory cortex (SI) (X = −4.2, Y = −2.2, Z = −0.2 in Paxinos space) and left hippocampus (Hp) (X = −4.8, Y = −2.4, Z = −0.4 in Paxinos space), two areas known to be involved in storage and recall of information and cognitive processing [44]. The time courses of seed region voxels were linearly de-trended to remove linear signal drift and averaged to create a single low-frequency reference time course. The preprocessing time courses were used as references and cross-correlated on a voxel basis across the whole brain to derive connectivity maps for each resting-state data set. The resulting correlation maps were overlaid on the standard rat brain template to exhibit the anatomical location of significant correlations. The dependence was quantitatively assessed by calculating the number of significant voxels (correlation threshold of *P*<2.5×10^−5^ with reference waveform, taking into account the reduced degrees of freedom in the low-pass filtered data) to detect areas of LFF higher than the global mean.

Cognitive testing data were analyzed by an observer blinded to the experimental protocol. Results are expressed as mean ±SD. Data were analyzed with one-way analysis of variance, *P*<0.05 being taken as statistically significant.

## Results

### Y Maze Results: Aversive Learning and Spatial Memory

**Figure 1:**
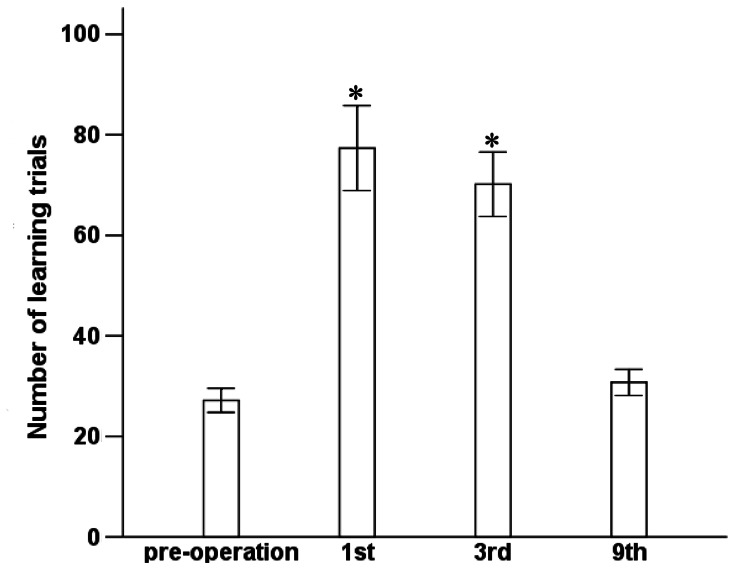
Learning and spatial memory for Y maze. Preoperatively and then 1, 3, 9 days after surgery, cohorts of rats were assessed in the Y maze apparatus to determine the trial number at which the rat first entered into the lit, unshocked arm for 9 out of 10 consecutive trial. Results are mean ± SD. *P<0.01 vs preoperative.

Preoperatively, it took 27±5 trials for aged rats to remember the risk of being shocked. On postoperative days 1 and 3 this increased significantly to 77±18 and 70±14 trials (*P*<0.01 vs preoperative), respectively, decreasing to a number not significantly different from preoperative on day 9 (*P* = 0.37; Fig. 1). Thus anaesthesia and surgery induced a temporary impairment in spatial memory.

### Physiological Measurements

There were no significant changes between the start and end of the scan in pH (7.4±0.02 vs 7.4±0.02), in *p*CO_2_ (28±2 vs 29±2 mmHg) or in *p*O_2_ (169±4 vs 168±2 mmHg) (all P>0.4).

### Resting-state Functional Connectivity Map

**Figure 2:**
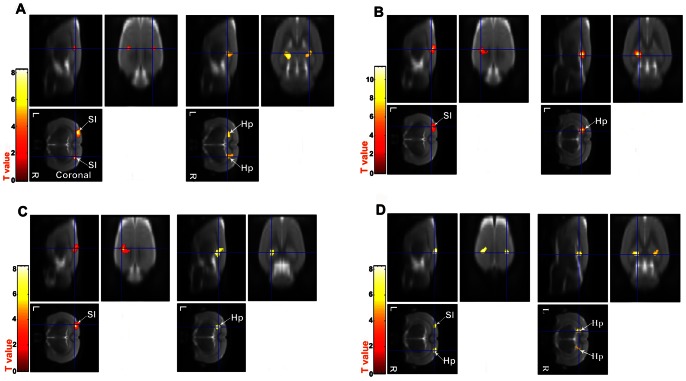
Functional connectivity assessed using fMRI by one sample *t*-test against 1 (*P*<0.001, uncorrected). SI, primary somatosensory cortex; Hp, hippocampus. L, left; R, right. Red and yellow means significantly synchronized LFF. (A) Preoperative (coronal slices): significant connectivity in bilateral SI and Hp. (B) Day 1 postoperative: functional connectivity virtually absent in right SI and right Hp. (C) Day 3 postoperative: functional connectivity still virtually absent in right SI and right Hp. (D) Day 9 postoperative: functional connectivity almost back to normal (cf panels A, B, C, D, respectively).

Excluding data from one rat which showed large nonlinear baseline drift, the results for 17 rats are shown in Fig. 2. Before operation (Fig. 2A) significant synchronized LFF (*P*<0.001, uncorrected) was detected bilaterally in the primary somatosensory cortex (SI) and hippocampus. By contrast, no significant LFF synchronization was detected in the right primary somatosensory cortex and right hippocampus on postoperative days 1 and 3 (Figs. 2B & 2C), although the functional connectivity pattern was almost normal by day 9 (Fig. 2D). This pattern was consistent for all rats. Table 1 and Table 2 show the number of significant voxels in the primary somatosensory cortex and hippocampus on postoperative days 1, 3 and 9, normalized by the preoperative value, and averaged seeds in the right primary somatosensory cortex and hippocampus respectively. The number of significant voxels is significantly decreased (as compared with preoperative) on postoperative days 1 and 3 (*P*<10^−9^ and *P*<10^−8^, respectively), but recovers by day 9.

**Table 1:** Significant voxels in the primary somatosensory cortex for the 1^st^, 3^rd^ and 9^th^ postoperative day, normalized by the total preoperative amount.

Rat	Preoperative	1st postoperative day	3rd postoperative day	9th postoperative day
1	1.00	0.01	0.02	0.96
2	1.00	0.01	0.01	0.96
3	1.00	0.01	0.01	0.98
4	1.00	0.02	0.01	0.95
5	1.00	0.01	0.02	0.99
6	1.00	0.11**	0.01	0.94
7	1.00	0.01	0.01	0.95
8	1.00	0.01	0.01	0.98
9	1.00	0.01	0.01	0.99
10	1.00	0.01	0.01	0.96
11	1.00	0.01	0.01	0.98
12	1.00	0.02	0.01	0.93
13	1.00	0.01	0.01	0.97
14	1.00	0.01	0.01	1.00
15	1.00	0.01	0.01	0.95
16	1.00	0.02	0.01	0.96
17	1.00	0.01	0.01	0.95
18	1.00	0.01	0.01	0.99
Mean		0.02(±0.02)	0.01(±0.00)	0.97(±0.02)

** Data from one rat which showed large nonlinear baseline drift.

**Table 2:** Significant voxels in the hippocampus for the 1^st^, 3^rd^ and 9^th^ postoperative day, normalized by the total preoperative amount.

Rat	Preoperative	1st postoperative day	3rd postoperative day	9th postoperative day
1	1.00	0.02	0.01	0.94
2	1.00	0.01	0.01	0.97
3	1.00	0.01	0.02	0.93
4	1.00	0.01	0.01	0.95
5	1.00	0.01	0.03	0.95
6	1.00	0.13**	0.01	0.91
7	1.00	0.02	0.01	0.95
8	1.00	0.01	0.01	0.99
9	1.00	0.03	0.02	0.92
10	1.00	0.01	0.01	0.93
11	1.00	0.01	0.02	0.95
12	1.00	0.02	0.01	0.95
13	1.00	0.01	0.02	0.98
14	1.00	0.01	0.02	0.95
15	1.00	0.01	0.01	0.96
16	1.00	0.02	0.01	0.95
17	1.00	0.01	0.02	0.97
18	1.00	0.02	0.01	0.98
Mean		0.02(±0.03)	0.01(±0.01)	0.95(±0.02)

** Data from one rat which showed large nonlinear baseline drift.

**Figure 3:**
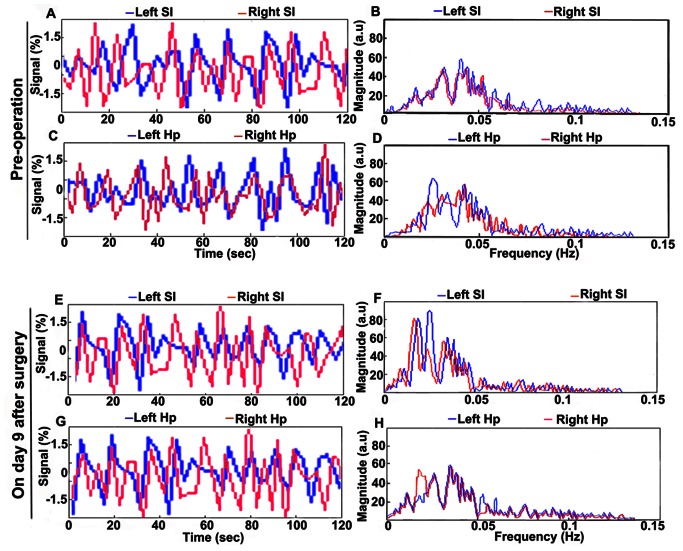
Comparisons of the timecourses from symmetrical ROIs in two hemispheres, and their Fourier Transforms. The timecourses from left-side (blue) and right-side (red) ROIs of SI (A & E) and Hp (C & G) are displayed as percentage signal fluctuations. Panels B, D, F and H are Fourier Transforms of panels A, C, E and G, respectively.

To demonstrate the synchronization of LFF in bilateral SIs preoperatively and on postoperative day 9, Figs. 3A & 3E show the timecourse of the seed ROI in the SI in one hemisphere compared with the average timecourse of the SI ROI in the opposite hemisphere; Fig. 3B & 3F show the Fourier Transforms of these timecourses. Similarly, Figs. 3C & 3G show the timecourses in the bilateral Hps preoperatively and on postoperative day 9; Figs. 3D & 3H show the Fourier Transforms. Significant correlation can be seen between the two timecourses.

## Discussion

Postoperative cognitive decline, a distressing complication after cardiac surgery or non-cardiac surgery, is independently associated with poor short- and long-term outcomes [8], [11], [45], [46]. Although its mechanisms remain unclear, hypoxemia, hypotension and embolism have often been cited [10]. Research has focused on neuropsychological and behavioral tests or pathological techniques 6,35,36,47. The present study may help to fill the gap between these by throwing light on neural function *in vivo*.

Evidence from both human and animal studies that some regions exhibiting task-related deactivation are functionally connected in the resting state [48], [49], suggests that synchronized LFF of the resting-state BOLD fMRI signal and the stimulus-induced BOLD signal may share the same underlying functional anatomy. We used synchronization of LFF to investigate the association of functional connectivity with the development in an animal model of cognitive impairment. Preoperatively, we found significant synchronized LFF in the primary somatosensory cortex (SI) and hippocampus (Hp), in agreement with previous studies [22]. The activity in SI, in particular suggests that resting state connectivity can identify the entire relevant network, as has been seen in animal and human studies [22], [50]. On days 1 and 3 after surgery under neuroleptic general anaesthesia, compared with the preoperative state, we found a lack of significant LFF synchronization in the right SI and right Hp, which however returned almost to normal by day 9.

Synchronized LFF of BOLD fMRI signals are believed to reflect functional connectivity, *viz.* spatiotemporal correlations between spatially remote neurophysiological events. Although the origin of synchronized LFF of BOLD has not been fully elucidated, some investigators attribute it to spontaneous neuronal activity [51]–[53]. Respiration and cardiac movements may also contribute to fluctuation of BOLD signals [54], via aliasing effects when their frequencies (∼1 Hz and ∼5 Hz, respectively, in rats [22]) exceed twice the sampling rate (0.5 Hz). It seems unlikely that the very different temporal patterns in the cortex and the Hp networks could arise mainly from a single central source.

In our study, to demonstrate the synchronized LFF in the BOLD signal in bilateral Sis preoperatively and on postoperative day 9, the timecourse of the seed ROI in the SI in one of the hemisphere is compared with the average timecourse of the SI ROI in the opposite hemisphere. As indicated in Figs. 3A & 3E, there is significant correlation between the two timecourses, demonstrating that LFF of BOLD signal in the bilateral SI in these rats are highly connected preoperatively and on postoperative day 9. Similarly, timecourse in the bilateral Hps are also observed, correlation between the two timecourses is evident by Fig. 3C & 3G.

During the scan we used chloral hydrate (300 mg/kg), administered intraperitoneally (requiring no catheterization) to produce temporary sedation and muscle relaxation [55], while maintaining the animal in a free breathing state requiring no intubation. This is similar to medetomidine, which is known not to influence functional connectivity at sedative levels [22]. Although an effect of chloral hydrate on functional connectivity cannot be ruled out, this was used as baseline for assessment of the postoperative data.

In our studies, the number of trials in the Y maze test was significantly increased on postoperative days 1 and 3, consistent with the changes in functional connectivity in the cortex and Hp. This is interesting given that the earliest functional manifestation of neuronal damage in the brain is a decline in the hippocampal and cortical functions of information storage and recall and cognitive processing [2], [44]. The underlying mechanism remains to be investigated. Although some investigators have attributed cognitive impairment to cellular apoptosis caused by perioperative factors such as hypoxia, hypocapnia [56], in the present study the respiration rate and oxygen saturation were monitored and maintained during surgery. Furthermore, synchronized LFF and spatial learning and memory are almost back to normal on day 9, which is hard to reconcile with cell death. What we have observed may be a temporary deficiency in spontaneous neuronal activity, induced by anaesthesia and surgery, in the right primary somatosensory cortex and hippocampus.

A limitation of this study is that it did not include a group that underwent anesthesia without surgery, or with sham surgery. Clearly further studies are needed to establish the causation and clinical relevance of our observation, including other anaesthetic regimens, and other types of surgery.

In conclusion, in this rat model of cognitive impairment, anaesthesia and surgery triggers a transient neurocognitive decline (apparent on postoperative days 1 & 3, back to normal at day 9), at the same time as reduced synchronization of temporal fMRI correlations in the SI and Hp. Splenectomy performed during neuroleptic anaesthesia triggers a cognitive decline that is associated with altered spontaneous neuronal activity in the cortex and hippocampus. The ability to detect noninvasively abnormalities of functional connectivity offers a way of exploring the mechanisms of postoperative cognitive dysfunction in patients.
